# Older age, higher BMI, female sex and meniscal repair are predictors of inferior patient‐reported outcomes 1 year after ACL reconstruction

**DOI:** 10.1002/ksa.12744

**Published:** 2025-06-29

**Authors:** Junya Tsukisaka, Atsuo Nakamae, Kazuhiro Tsukisaka, Ryo Shimizu, Yuki Yamanashi, Akinori Nekomoto, Kyohei Nakata, Nobuo Adachi

**Affiliations:** ^1^ Department of Orthopaedic Surgery Graduate School of Biomedical and Health Sciences Hiroshima University Hiroshima Japan; ^2^ Department of Orthopaedic Surgery Mazda Hospital Hiroshima Japan; ^3^ Department of Orthopaedic Surgery JA Onomichi General Hospital Hiroshima Japan; ^4^ Department of Orthopaedic Surgery Aichi Medical University Hospital Aichi Japan

**Keywords:** anterior cruciate ligament (ACL), knee, multi‐centre study, patient‐reported outcomes (PROs), predictors

## Abstract

**Purpose:**

To identify predictors of inferior patient‐reported outcomes (PROs) 1 year after anterior cruciate ligament reconstruction (ACLR) and to examine whether these predictors differ by sex. We hypothesised that older age, female sex, and meniscectomy are associated with inferior PROs.

**Methods:**

We conducted a prospective cohort study involving 733 patients (396 males and 337 females) who underwent ACLR using autologous hamstring tendons. Participants were enroled in the Hiroshima Clinical ACL Research Project. Pre‐operative assessments included demographic data, injury history, and scores from the five subscales of the Knee Injury and Osteoarthritis Outcome Score (KOOS). Additional assessments included the International Knee Documentation Committee (IKDC) subjective form, Lysholm score, and Tegner activity scale. Multivariable linear regression analysis was performed to identify predictors of KOOS subscale outcomes, including sex‐specific subgroup analyses at 1 year postoperatively.

**Results:**

Older age was a significant predictor of poorer KOOS outcomes across all subscales (*p* < 0.001). Higher body mass index (BMI) was also associated with inferior KOOS outcomes overall (*p* = 0.000–0.012). Female sex was significantly associated with worse outcomes on the KOOS Symptom (*p* = 0.003), Pain (*p* = 0.001), ADL (*p* = 0.002), and Sports/Recreation (*p* = 0.001) subscales. In female patients, higher BMI was significantly associated with all KOOS subscales (*p* = 0.000–0.007), while no such associations were observed in males. Medial meniscal repair was associated with significantly poorer KOOS Symptom (*p* < 0.001), Sports/Recreation (*p* < 0.001), and QOL (*p* = 0.018) scores. Lateral meniscal repair was significantly associated with worse KOOS Sports/Recreation (*p* = 0.003) and QOL (*p* = 0.005) scores. Contrary to our hypothesis, meniscectomy was not a significant predictor (*p* > 0.05).

**Conclusion:**

Older age, higher BMI, female sex, and meniscal repair were predictors of inferior PROs 1 year after ACLR. These findings underscore the importance of sex‐specific preoperative counselling and postoperative management for patients at higher risk of suboptimal outcomes.

**Level of Evidence:**

Level II.

**Abbreviations**
ACL: anterior cruciate ligament
ACLR: anterior cruciate ligament reconstruction
BMI: body mass index
FTA: femoro‐tibial angle
IKDC: international knee documentation committee
KOOS: knee injury and osteoarthritits outcome score
PROs: patient‐reported outcomes
QOL: quality of life
ROM: range of motion

## INTRODUCTION

Anterior cruciate ligament (ACL) rupture is a common sports‐related injury that increases anterior and rotational knee laxity, leading to instability and reduced function, particularly in young, active individuals [1]. ACL reconstruction (ACLR) is among the most frequently performed orthopaedic procedures [19] and ACLR has proven effective in reducing anterior and rotational instability [2, 3, 4].

Postoperative patient‐reported outcomes (PROs) at 1 year have been reported to correlate with physical function in patients undergoing ACL reconstruction [25]. Several factors have been associated with PROs; however, these remain a subject of debate. Hamrin has reported that younger age, male sex, nonsmoking status, use of hamstring tendons for surgery and the absence of concomitant injuries are associated with better PROs [14]. Several studies have indicated that concomitant meniscectomy at the time of ACL reconstruction may lead to inferior postoperative outcomes, including increased clinical symptoms and radiographic signs of degeneration [16, 27]. Previous studies suggest that meniscal repair, rather than meniscectomy, is a significant predictor of long‐term PROs [7].

Despite this, predictors of PROs remain inadequately identified. This study aimed to determine the predictors of inferior PROs 1 year after ACLR. We hypothesised that older age, female sex, and meniscectomy would be associated with inferior PROs. Furthermore, in response to recent concerns regarding sex‐specific differences in clinical outcomes, we additionally performed separate analyses for male and female patients to explore whether the predictors of inferior PROs differ between sexes.

## MATERIALS AND METHODS

This study was approved by the Institutional Review Board of Hiroshima University Hospital (ID number: E2018‐1178) and the institutional review board of all participating sites.

The patients included in this study were enroled in the Hiroshima Clinical ACL Research Project (Hiroshima CARP), an ongoing prospective multicentre cohort study involving individuals who underwent ACL reconstruction. All patients underwent primary ACL reconstruction using autologous hamstring tendons, performed by 12 surgeons across ten participating institutions. One hospital served as the data processing centre and was responsible for data collection. The recruitment period for the study was between January 2018 and December 2020. The inclusion criteria were as follows: normal contralateral knee, no history of surgery on the ipsilateral knee, and no posterior cruciate ligament insufficiency. Patients with revision ACLR, discoid lateral meniscus, grade III medial or lateral instability, or congenital ACL deficiency were excluded. This study placed no restrictions on lower‐extremity alignment. All participating surgeons had completed a specialised training programme in knee arthroscopy at Hiroshima University before joining the study. The training ensured consistency in performing physical examinations, including the pivot shift and Lachman tests.

### Data sources and measurement

Data were collected from a prospective multicentre cohort of patients who underwent ACL reconstruction using autologous hamstring tendons and were followed up 1 year after surgery. Each patient underwent an arthrometer assessment and pivot‐shift test and completed PROs assessments, including the five subscales of the knee injury and osteoarthritis outcome score (KOOS) [33], International Knee Documentation Committee (IKDC) subjective form [15], Lysholm knee scale, and Tegner activity score [6]. Assessments were conducted both pre‐operatively and 1 year post‐operatively. Patients were also required to complete an enrolment form to provide detailed information on demographics, injury characteristics, sports participation history, occupation, duration between ACL injury and surgery, physical activity levels before injury and surgery, comorbidities, and previous knee surgeries on either knee.

Surgeons were required to use standardised evaluation forms to document their findings on the pivot shift test, Lachman test, knee range of motion (ROM), and varus and valgus knee instability. The pivot‐shift test was graded according to the IKDC criteria as follows: 0 (equal), 1 + (glide), 2 + (clunk) or 3 + (gross). In addition to the conventional grades of the pivot shift test, patients' subjective apprehension during the test was also classified as 0 (no apprehension), 1 + (mild apprehension), 2 + (moderate apprehension), or 3 + (severe apprehension) [22, 24]. A grade of ≥1 on the pivot‐shift test, based on either IKDC or apprehension grading, was defined as positive, while a grade of 0 was defined as negative. Instrumented knee laxity testing devices were used to measure both the absolute values and inter‐limb differences in anterior knee laxity. Bilateral standing full‐length radiographs were obtained pre‐operatively to determine the femorotibial angle (FTA). The Kellgren–Lawrence classification system was employed to assess the presence and severity of knee osteoarthritis radiographically.

During reconstruction, the operating surgeon documented arthroscopic findings and surgical techniques, including graft type, as well as meniscal and cartilage pathology and treatment. Meniscal status was classified as normal, stable meniscal tear without treatment, meniscal repair, or partial meniscectomy. The medial and lateral menisci were graded as 0 (normal), 1 (minor damage), 2 (major damage, excluding bucket handle tears), or 3 (bucket handle tears). Grade 1 (minor damage) referred to a stable meniscus with minor damage that did not require repair or resection. Grade 2 (major damage) represented an unstable meniscus requiring repair or resection. For subsequent evaluations, grades 0 and 1 were classified as ‘normal’ meniscal conditions, and Grades 2 and 3 were classified as ‘damaged’ meniscal conditions. The condition of the articular cartilage was assessed at the following regions: the medial femoral condyle and medial tibial plateau (medial compartment), lateral femoral condyle and lateral tibial plateau (lateral compartment), and patella and femoral trochlea (patellofemoral [PF] compartment). Particular attention was given to the presence of concomitant injuries. Surgeons completed standardised evaluation forms documenting detailed aspects of the treatment, including surgical techniques (single‐bundle reconstruction, double‐bundle reconstruction, or remnant‐preserving single‐bundle ACL augmentation), graft size, femoral tunnel length, femoral tunnel drilling technique, graft fixation methods, meniscal condition or treatment (normal, meniscal repair, or partial meniscectomy), and articular cartilage conditions.

### Rehabilitation protocol

Patients without meniscal injury, or those who underwent meniscectomy despite the presence of a meniscal injury, were immobilised for the first three postoperative days. Thereafter, range of motion (ROM) exercises were initiated using a soft ACL brace. Partial weight‐bearing was permitted from approximately postoperative Week 1, with progression to full weight‐bearing between Weeks 3 and 4 depending on pain tolerance. For patients who underwent meniscal repair, immobilisation was maintained for 1–2 weeks postoperatively. Subsequently, ROM exercises were initiated under protection with a soft ACL brace. Partial weight‐bearing was allowed between postoperative Weeks 2 and 4 depending on the extent of the meniscal injury, followed by progression to full weight‐bearing between Weeks 5 and 7. Across all participating centres, an ACL brace was used for 2–3 months postoperatively, and return to sports was generally permitted between 9 and 12 months after surgery.

### Statistical analysis

Statistical analyses were conducted using SPSS Statistics for Windows (version 22.0; IBM Corp., Armonk, New York, USA). Patient characteristics are summarised as the mean ± standard deviation, number of patients, and/or frequency (%).

Comparisons between the pre‐operative and post‐operative data were made using the paired *t*‐test or Wilcoxon signed‐rank test. Multiple linear regression analysis with stepwise backward selection was employed to identify potential predictors of inferior PROs 1 year after ACLR. Potential predictors included age, sex, duration between injury and surgery, body mass index (BMI), meniscal condition or treatment (normal, meniscal repair, or partial meniscectomy), articular cartilage condition, pivot‐shift test grade, anterior knee laxity, Tegner activity scale score before injury, five KOOS subscales before surgery, FTA, knee ROM before surgery, and ACL reconstruction procedures (single‐bundle reconstruction, double‐bundle reconstruction, or remnant‐preserving single‐bundle reconstruction). *p*‐Value < 0.05 was considered statistically significant.

## RESULTS

Overall, 733 participants were included in this study: 397 males (51.4%) and 376 females (48.6%) (Table 1). Pre‐operative and post‐operative clinical outcomes are presented in Table 2. Multiple linear regression analyses indicated that older age at ACL and higher BMI were significant predictors of poor PROs in all five KOOS subscales (*p* < 0.001 and *p* = 0.000–0.012, respectively) (Table 3). Being female was significantly associated with inferior PROs on four KOOS subscales: KOOS Symptom (*p* = 0.003), Pain (*p* = 0.001), Activities of Daily Living (*p* = 0.002), and Sports/Recreation (*p* = 0.001) (Table 3). Medial meniscal repair significantly influenced inferior PROs on KOOS Symptom (*p* < 0.001), Sports/Recreation (*p* < 0.001), and Quality of Life (*p* = 0.018) subscales. Lateral meniscal repair was associated with inferior PROs on KOOS Sports/Recreation (*p* = 0.003) and Quality of Life (*p* = 0.005) subscales (Table 3). In male patients, older age and lateral meniscal repair were associated with inferior KOOS outcomes (*p* = 0.000–0.046), while lower preoperative KOOS scores also predicted inferior results (*p* = 0.000–0.028). BMI showed no significant association with any KOOS subscale (n.s.) (Table 4). Otherwise, in female patients, older age, higher BMI, and medial meniscal repair were associated with inferior KOOS outcomes (*p* = 0.000–0.020), while lower preoperative KOOS Pain scores predicted inferior Pain and ADL results (*p* =0 .001 and *p* = 0.010, respectively) (Table 5). With respect to the differences in postoperative KOOS subscales among the participating institutions, no significant differences were observed among the 10 institutions (n.s.). Moreover, regarding the surgical methods, no significant differences in any KOOS subscales were observed among the three surgery groups after surgery (n.s.).

**Table 1:** Patient characteristics.

		Total	Male	Female	*p*‐value
Number of patients		773	397	376	–
Mean age (years)		28.8 ± 12.9	30.3 ± 12.1	27.2 ± 13.5	**0.001**
Mean BMI at surgery (kg/m^2^)		24.0 ± 4.0	24.8 ± 4.1	23.1 ± 3.8	**0.000**
Mean duration between injury and surgery (months)		16.8 ± 51.4	15.6 ± 12.9	18.0 ± 53.4	0.510
Mean femorotibial angle (degree)		175.2 ± 3.3	175.7 ± 3.4	174.8 ± 3.1	0.401
Mean side‐to‐side difference of anterior knee laxity before ACL reconstruction (mm)		3.3 ± 2.6	3.3 ± 2.5	3.2 ± 2.7	0.674
Pivot‐shift phenomena before surgery (IKDC grading) (Grade 0: 1: 2: 3)		74: 434: 218: 47	38: 243: 98: 18	36: 191: 120: 29	**0.015**
Apprehension grading of the pivot‐shift test before surgery (Grade 0: 1: 2: 3)		88: 383: 255: 47	42: 215: 124: 16	46: 168: 131: 31	**0.016**
Mean Tegner activity level scale before injury		6.5 ± 1.7	6.6 ± 1.6	6.4 ± 1.7	**0.026**
Mean preoperative Lysholm knee score (points)		71.6 ± 19.2	71.7 ± 19.1	71.6 ± 19.3	0.913
Mean preoperative IKDC score (points)		57.6 ± 17.0	57.2 ± 17.6	58.0 ± 16.3	0.546
Mean preoperative KOOS Symptom (points)		74.9 ± 19.1	73.4 ± 19.1	76.5 ± 18.9	**0.023**
Mean preoperative KOOS Pain (points)		76.6 ± 18.4	76.2 ± 18.8	77.1 ± 18.0	0.495
Mean preoperative KOOS ADL (points)		85.7 ± 16.3	84.5 ± 17.1	87.0 ± 15.4	**0.033**
Mean preoperative KOOS Sports (points)		49.2 ± 28.6	49.4 ± 29.6	48.9 ± 27.5	0.796
Mean preoperative KOOS QOL (points)		47.8 ± 25.1	45.8 ± 25.1	50.0 ± 25.0	**0.018**
ACL reconstruction procedures	Single‐bundle reconstruction	336	159	177	**0.032**
	Double‐bundle reconstruction	162	97	65	
	Remnant‐preserving single‐bundle augmentation	275	141	134	
Treatments for the meniscus (None: Resection: Repair)	Medial meniscus	539: 93: 141	289: 45: 63	250: 48: 78	0.139
	Lateral meniscus	582: 97: 94	284: 58: 55	298: 39: 39	**0.045**
Articular cartilage conditions (Normal: Damaged)	Medial compartment	558: 215	282: 115	276: 100	0.256
	Lateral compartment	605: 168	313: 84	292: 84	0.378
	Patellofemoral compartment	658: 115	345: 52	313: 63	0.092

*Note*: Values are reported as mean ± SD, %, or number of patients.

Abbreviations: ACL, anterior cruciate ligament; BMI, body mass index; IKDC, International Knee Documentation Committee; KOOS, Knee Injury and Osteoarthritis Outcome Score; QOL, Quality of Life; SD, standard deviation.

**Table 2:** One year postoperative clinical outcomes.

	Total	Male	Female	*p*‐value
Mean side‐to‐side difference of anterior knee laxity (mm)	0.8 ± 2.3	0.7 ± 2.3	0.9 ± 2.3	0.354
Pivot‐shift phenomena (IKDC grading) (Grade 0: 1: 2: 3)	652: 95: 26: 0	342: 40: 15: 0	310: 55: 11: 0	0.136
Apprehension grading of the pivot‐shift test (Grade 0: 1: 2: 3)	664: 92: 17: 0	353: 38: 6: 0	311: 54: 11: 0	0.042
Mean Lysholm knee score (points)	93.7 ± 10.1	94.0 ± 10.6	93.3 ± 9.5	0.351
Mean IKDC score (points)	81.9 ± 14.2	82.9 ± 14.1	80.8 ± 14.3	0.045
Mean KOOS Symptom (points)	88.1 ± 13.2	88.4 ± 12.1	87.7 ± 14.3	0.494
Mean KOOS Pain (points)	92.5 ± 9.7	92.9 ± 9.2	92.1 ± 10.3	0.206
Mean KOOS ADL (points)	97.4 ± 5.2	97.6 ± 4.8	97.2 ± 5.6	0.286
Mean KOOS Sports (points)	85.3 ± 16.9	85.8 ± 15.9	84.8 ± 18.0	0.416
Mean KOOS QOL (points)	80.2 ± 18.7	79.4 ± 19.1	81.1 ± 18.3	0.200

*Note*: Values are reported as mean ± SD, %, or number of patients.

Abbreviations: IKDC, International Knee Documentation Committee; KOOS, Knee Injury and Osteoarthritis Outcome Score; QOL, Quality of Life; SD, standard deviation.

**Table 3:** Significant risk factors for poor postoperative KOOS subscores 1 year after ACL reconstruction in multivariable linear regression model.^a^

	Postoperative KOOS symptom		Postoperative KOOS pain		Postoperative KOOS ADL		Postoperative KOOS sports		Postoperative KOOS QOL	
Factors	*β* (95% CI of B)	*p*‐value	*β* (95% CI of B)	*p*‐value	*β* (95% CI of B)	*p*‐value	*β* (95% CI of B)	*p*‐value	*β* (95% CI of B)	*p*‐value
Age	−0.27 (−0.35 to −0.21)	0.000	−0.22 (−0.22 to −0.11)	0.000	−0.23 (−0.12 to −0.06)	0.000	−0.24 (−0.4 to −0.22)	0.000	−0.22 (−0.42 to −0.27)	0.000
BMI	−0.09 (−0.52 to −0.07)	0.012	−0.13 (−0.49 to −0.14)	0.000	−0.15 (−0.28 to −0.10)	0.000	−0.16 (−0.98 to −0.40)	0.000	−0.10 (−0.80 to −0.16)	0.004
Female	0.10 (0.93 to 4.5)	0.003	0.11 (0.86 to 3.6)	0.001	0.11 (0.43 to 1.89)	0.002	0.11 (1.5 to 6.1)	0.001	–	–
Medial meniscal repair	0.15 (2.8 to 7.3)	0.000	–	–	–	–	0.12 (2.4 to 8.2)	0.000	0.08 (0.66 to 7.1)	0.018
Lateral meniscal repair	–	–	–	–	–	–	0.10 (1.8 to 8.6)	0.003	0.10 (1.7 to 9.4)	0.005
Inferior preoperative KOOS QOL	–	–	–	–	–	–	0.23 (0.11 to 0.20)	0.000	0.26 (0.15 to 0.25)	0.000

Abbreviations: ADL, activities of daily living; *β*, standardised partial regression coefficient; B, partial regression coefficient; BMI, body mass index; CI, confidence interval; KOOS, Knee Injury and Osteoarthritis Outcome Score; QOL, Quality of Life.

a This table lists the significant risk factors that were present in two or more of the five subscores of the KOOS.

**Table 4:** Significant risk factors for poor KOOS subscores one year after ACL reconstruction in male patients (multivariable linear regression model).^a^

	Postoperative KOOS symptom		Postoperative KOOS pain		Postoperative KOOS ADL		Postoperative KOOS Sports		Postoperative KOOS QOL	
Factors	*β* (95% CI of B)	*p*‐value	*β* (95% CI of B)	*p*‐value	*β* (95% CI of B)	*p*‐value	*β* (95% CI of B)	*p*‐value	*β* (95% CI of B)	*p*‐value
Age	−0.27 (−0.35 to −0.16)	0.000	−0.14 (−0.18 to −0.03)	0.006	−0.20 (−0.12 to −0.04)	0.000	−0.19 (−0.37 to −0.12)	0.000	−0.13 (−0.37 to −0.05)	0.011
Lateral meniscal repair	−0.10 (−6.60 to −0.05)	0.046					−0.17 (−11.8 to −3.1)	0.001	−0.17 (−15.0 to −4.0)	0.001
Inferior preoperative KOOS ADL	–	–	0.26 (0.08 to 0.19)	0.000	0.24 (0.04 to 0.09)	0.000			–	–
Inferior preoperative KOOS QOL	–	–	–	–	–	–	0.25 (0.09 to 0.21)	0.000	0.29 (0.15 to 0.30)	0.000

Abbreviations: ADL, activities of daily living; *β*, standardised partial regression coefficient; B, partial regression coefficient; BMI, body mass index; CI, confidence interval; KOOS, Knee Injury and Osteoarthritis Outcome Score; QOL, Quality of Life.

a This table lists the significant risk factors that were present in two or more of the five subscores of the KOOS.

**Table 5:** Significant risk factors for poor KOOS subscores one year after ACL reconstruction in female patients (multivariable linear regression model).^a^

	Postoperative KOOS symptom		Postoperative KOOS pain		Postoperative KOOS ADL		Postoperative KOOS sports		Postoperative KOOS QOL	
Factors	*β* (95% CI of B)	*p*‐value	*β* (95% CI of B)	*p*‐value	*β* (95% CI of B)	*p*‐value	*β* (95% CI of B)	*p*‐value	*β* (95% CI of B)	*p*‐value
Age	−0.27 (−0.39 to −0.18)	0.000	−0.22 (−0.25 to −0.09)	0.000	−0.30 (−0.17 to −0.08)	0.000	−0.30 (−0.52 to −0.27)	0.000	−0.25 (−0.48 to −0.19)	0.000
BMI	−0.19 (−1.06 to −0.34)	0.000	−0.24 (−0.90 to −0.38)	0.000	−0.21 (−0.45 to −0.17)	0.000	−0.19 (−1.35 to −0.45)	0.000	−0.13 (−1.08 to −0.18)	0.007
Medial meniscal repair	−0.19 (−10.2 to −3.6)	0.000	−0.11 (−5.27 to −0.46)	0.020			−0.13 (−10.2 to −1.8)	0.005		
Inferior preoperative KOOS pain	–	–	0.17 (0.04 to −0.15)	0.001	0.13 (0.01 to −0.07)	0.010				

Abbreviations: ADL, activities of daily living; *β*, standardised partial regression coefficient; B, partial regression coefficient; BMI, body mass index; CI, confidence interval; KOOS, Knee Injury and Osteoarthritis Outcome Score; QOL, Quality of Life.

a This table lists the significant risk factors that were present in two or more of the five subscores of the KOOS.

## DISCUSSION

The most significant finding of this study was that older age, higher BMI, female sex, and meniscal repair were identified as predictors of inferior PROs 1 year after ACLR. These findings partially support the primary hypothesis of this study. While older age and female sex were confirmed as predictors of inferior PROs, meniscectomy was not associated with inferior outcomes. Instead, medial and lateral meniscal repair emerged as significant predictors of inferior PROs, influencing outcomes on three and two KOOS subscales, respectively.

In this study, older age was associated with inferior KOOS scores across all five subscales. While not directly comparing with younger patients, Pohl et al. investigated postoperative outcomes between patients aged ≥60 years and those aged 40–59 years undergoing ACL reconstruction, and reported comparable clinical results [28]. In contrast, previous research has indicated that younger age, male sex, and the absence of concomitant injuries are associated with superior PROs after ACL reconstruction [8, 14]. Younger patients tend to report better KOOS scores both pre‐ and post‐operatively. However, during follow‐up periods, older patients (≥40 years) reported greater relative improvements in KOOS scores. This suggests that older patients may perceive more substantial benefits from ACL reconstruction than younger patients, although this perception could be influenced by factors such as physical activity levels and motivation to return to sports [14]. Miyamoto et al reported that patients aged ≥40 years scored the Tegner activity level scale and IKDC score lower from before surgery to 1 year after surgery than the patients aged ≤20 years [20]. Therefore, the younger cohort had a higher level of functional demand in their daily activities compared to the older cohort. Furthermore, the younger group demonstrated a greater inclination towards returning to sports, engaged in appropriate training, and consequently experienced a more favourable recovery.

A higher BMI was also associated with inferior KOOS scores across all five subscales. A recent study conducted by the MARS group found that higher BMI increased the risk of articular cartilage deterioration in the PF compartment during revision ACL reconstruction [13]. These findings suggest that elevated BMI may have the greatest impact on the PF compartment. When PF joint damage occurs alongside an ACL injury, it can contribute to a decline in PROs. Additionally, the higher BMI was identified as a significant predictor of inferior postoperative outcomes in female patients, whereas no such association was observed in male patients. Previous studies have reported that higher BMI is correlated with increased collagen turnover on return to activity, which may reflect alterations in tissue remodelling processes [29]. Additionally, higher BMI, lower education level, smoking, and revision ACL surgery have been identified as significant predictors of inferior KOOS scores [8]. Moreover, higher type II collagen turnover ratios, which have been shown to be more pronounced in females, are associated with abnormal joint loading and an increased risk of joint space narrowing following ACL reconstruction [18]. These findings suggest that the combination of elevated BMI and sex‐specific differences in collagen metabolism may contribute to inferior outcomes in female patients after ACL reconstruction.

In the present study, the female sex was identified as a predictor of inferior outcomes on all KOOS subscales except for quality of life (QOL). Females exhibited fewer improvements than males on the KOOS Sport and Recreation and Symptoms subscales over time after ACLR [5]. Regarding IKDC scores among PROs, Mok et al. reported that male patients achieved significantly higher post‐operative IKDC scores than females [21]. These differences in PROs suggest that males and females follow distinct long‐term recovery trajectories after ACLR. Kidwell et al. reported that females demonstrated lower knee function self‐efficacy and psychological readiness to return to sports at 6 months after ACLR [11]. Additionally, Grand et al. suggested that female adolescents were better able to accurately identify and classify the presence or absence of ACL injury compared to males [12]. Moreover, Sun et al. indicated that female sex is a poor predictor of the patient acceptable symptom state (PASS), minimal clinically important difference (MCID) and minimal important change (MIC) for PROs following ACLR [31]. Ebert et al. have demonstrated that female sex reported less postoperative activity and psychological readiness to return to sports over the first 2 postoperative years and also lower the Anterior Cruciate Ligament Return to Sport after Injury scores at 12 and 24 months [10]. Cristiani et al. also observed that being female (vs. male) reduced the odds of reaching a PASS on the KOOS subscales [9]. These cumulative findings suggest that female patients may experience greater psychological and functional challenges throughout the recovery process following ACL reconstruction, which may contribute to inferior PROs.

Meniscal injury is a significant predictor of poor KOOS scores, as it is negatively associated with PROs [14]. There is no correlation between preexisting osteoarthritic changes or chondral defects and PROs [17]. During rotational subluxation of the lateral compartment at the time of ACL injury, energy is transferred to either the meniscus or the articular cartilage [8]. Additionally, persistent severe anterior knee instability after ACL injury can contribute to meniscal damage [23]. Svantesson et al. reported that patients in both medial and lateral meniscal repair groups had lower PRO scores compared to those in the isolated ACLR group at the 6‐month follow‐up [32]. Post‐operative management of meniscal repairs is typically more restrictive than that of isolated ACL reconstructions or meniscal resections. This caution stems from concerns that weight‐bearing activities and knee flexion may lead to gapping of the meniscus, causing excessive tension and hoop stress [26, 30]. Our study, focusing on short‐term follow‐up, revealed results consistent with those of the current study. However, in long‐term follow‐up, patients with concomitant meniscal tears who underwent meniscal repair demonstrated improved PROs compared to those treated with meniscectomy [7]. Cox et al. also reported that both articular cartilage injury and meniscal tears or treatment at the time of ACLR were significant predictors of IKDC and KOOS scores 6 years after ACLR [8]. These findings suggest that, with long‐term follow‐up, meniscal repair may be associated with improved PROs.

The strengths of this study include the large sample size and its design as an ongoing, prospective, longitudinal multicentre cohort study. However, several limitations must be acknowledged. The primary limitation is the lack of clarity regarding the cause and timing of meniscal injuries. Additionally, the specific patterns of meniscal tears, such as horizontal or radial tears, were not clearly identified. As this study evaluated outcomes only at 1 year postoperatively, the long‐term effects of meniscectomy on PROMs and return to sports could not be fully assessed. Further follow‐up of this cohort is ongoing, and the long‐term results will be reported in future studies. Finally, this study included only patients who elected to undergo surgical treatment, which may limit the generalisability of the findings to the broader population of patients with ACL injuries.

## CONCLUSION

Older age and higher BMI were identified as predictors of inferior outcomes across all the KOOS subscales. Female sex was a predictor of poor outcomes for all KOOS subscales except for KOOS QOL. Medial meniscal repair was associated with inferior outcomes on the KOOS Symptom, Sports/Recreation, and QOL subscales, while lateral meniscal repair predicted inferior outcomes on the KOOS Sports/Recreation and QOL subscales. The clinical relevance of this study lies in its potential to enable healthcare providers to offer more appropriate informed consent to patients with predictors of inferior PROs. Furthermore, these findings underscore the importance of careful post‐operative monitoring and follow‐ups by medical professionals to address and manage these risk factors effectively.

## AUTHOR CONTRIBUTIONS

Atsuo Nakamae, Junya Tsukisaka, and Nobuo Adachi were involved in concept creation, data collection, data analysis, construction of the first draft of the manuscript and editing of the final manuscript. Kazuhiro Tsukisaka, Ryo Shimizu, Yuki Yamanashi, Akinori Nekomoto, and Kyohei Nakata were involved in concept creation and data collection.

## CONFLICT OF INTEREST STATEMENT

The authors declare no conflicts of interest.

## ETHICS STATEMENT

The current study was reviewed and approved by each participating site's respective institutional review board (Code; E2018‐1178), and informed consent was obtained from all participants included in this study.

## Data Availability

The data that support the findings of this study are available on request from the corresponding author, A.N. The data are not publicly available due to restrictions of their containing information that could compromise the privacy of research participants.
